# Variation in Soluble Sugars in Arabica Coffee Cherry Fruits

**DOI:** 10.3390/plants13131853

**Published:** 2024-07-05

**Authors:** João Leonardo Corte Baptistella, Giovane Assoni, Marcio Souza da Silva, Paulo Mazzafera

**Affiliations:** 1 Department of Crop Science, “Luiz de Queiroz” College of Agriculture, Esalq-Usp, Piracicaba 13418-900, Brazil; joao.baptistella@usp.br (J.L.C.B.); giovane.assoni@usp.br (G.A.); mssmarcio@yahoo.com (M.S.d.S.); 2 Department of Plant Biology, Institute of Biology, Campinas State University, São Paulo 13083-862, Brazil

**Keywords:** coffee quality, sucrose, *Coffea arabica*

## Abstract

The maturation of Arabica coffee fruits is influenced by both endogenous and external factors. The stage of fruit maturation affects the chemical composition of the beans, which in turn impacts the quality of the coffee beverage. During maturation, the fruit peel changes colour from green to red (cherry), signalling the optimal harvest time and suggesting high fruit quality. However, the degree of redness can vary, indicating different levels of maturity. This study aimed to explore the variation in soluble sugar accumulation in relation to the redness of coffee fruit tissues. We classified ripe fruits into six ripeness categories based on the intensity of the red colour of the epicarp, measured using a colourimeter. We analysed total soluble sugar, sucrose, and starch in three parts: coat (exocarp + mesocarp), coat juice (obtained by squeezing the coat), and beans. Our findings reveal that the variation in sugar in the endosperm does not correspond to changes in the coat, suggesting separate regulation of sugar accumulation, particularly sucrose, which is crucial for coffee quality. Our data indicate that there is no transfer of sucrose and reducing sugars from the red coat to the bean.

## 1. Introduction

The maturation of Arabica coffee fruits (*Coffea arabica* L.) is influenced by factors related to plant genetics, edaphoclimatic conditions, and their interactions [1]. Coffee variety, plant age, and physiology strongly impact bean quality. For instance, different varieties have varying maturation cycles, from early to late. The position of the fruits within the plant canopy also affects maturation, as the source–sink balance differs among fruits, and they experience varying light and temperature conditions based on their location [2]. Climate, altitude, and their regulatory effects on mean air temperature influence the maturation rate and the plant’s water demand, both of which are related to quality [3].

Immature and overripe beans are directly linked to lower coffee quality due to their distinct physicochemical properties and chemical compositions compared to ripe cherries [4,5]. Their presence in a coffee lot is considered a defect in the international coffee evaluation system. Immature coffee beans are lighter, more acidic, and have lower concentrations of sucrose, oil, and protein than mature beans [4]. Overripe fruits are susceptible to microbial attacks, leading to the development of undesirable products from microbial metabolism [5]. Therefore, maximizing the harvest of ripe fruits while minimizing immature and overripe fruits is crucial for maintaining high coffee beverage quality [1,4].

The chemical composition of coffee beans influences the beverage’s aroma, flavour, sourness, and sweetness. These sensory parameters result from a complex process of post-harvest and roasting reactions [6]. Among the various compounds in coffee beans, sucrose is a key precursor of coffee aroma and flavour [7,8].

*C. arabica* fruits exhibit a bimodal growth curve [9]. In the first growth phase, the endosperm is fully formed and the fruit reaches its full size. The second phase is when maturation occurs. Unlike proteins, alkaloids (caffeine and trigonelline) and most chlorogenic acids in the endosperm, which peak in concentration during the first growth phase [10], sucrose continues to accumulate until the end of maturation [9,11,12]. Sucrose also accumulates in the endosperm following a bimodal curve, reaching approximately 8% in mature fruit endosperm [12,13,14]. This increase during maturation is likely supported by sucrose imported from the leaves, although the contribution of epicarp photosynthesis to this sucrose increase remains uncertain [9]. During bean roasting, sucrose reacts with amino acids, peptides, and proteins in the Maillard reaction, forming new compounds. The Maillard reaction is responsible for developing coffee’s organoleptic attributes [7,8]. Therefore, the sucrose concentration in beans is of great importance for the final coffee quality. It is believed that the highest sucrose content is found in mature fruits, which, according to coffee farmers, is indicated by the fruit colour.

During maturation, the metabolism of coffee fruits changes markedly, and the colour of the fruit peel (epicarp) changes from green to red or yellow, depending on the cultivar. Coffee maturation can be classified into five stages based on the colour of the fruit peel: green (immature fruits), yellowish (semimature fruits), cherry (ripe fruits), dark red or dark yellow (overripe fruits), and black (dry fruits) [15,16]. In red fruit varieties, cherry fruits should be entirely red when they are physiologically mature and supposedly at their highest quality. Therefore, colour is closely linked to coffee quality, indicating the maturation stage and proper harvest timing for farmers.

The relationship between chemical composition and sensory characteristics plays a crucial role in defining coffee quality, with the ripe stage, characterized by red fruits, considered ideal for harvesting. At this point, coffee beans have completed their maturation, and sucrose, a vital component of coffee quality, has reached its peak concentration. However, ripe coffee fruits exhibit various shades of red. In this study, we investigated the variation in soluble sugar accumulation in relation to fruit redness in coffee fruit tissues. Ripe fruits were classified into six ripeness categories based on the intensity of the red colour of the pericarp using a colourimeter. Total soluble sugar, sucrose, and starch were analysed in the pericarp, pericarp juice, and beans. Our findings indicate that changes in the pericarp (exocarp + mesocarp) are not mirrored by the endosperm, suggesting separate control of sugar accumulation, particularly sucrose, a critical component of coffee quality.

## 2. Results

**Figure 1:**
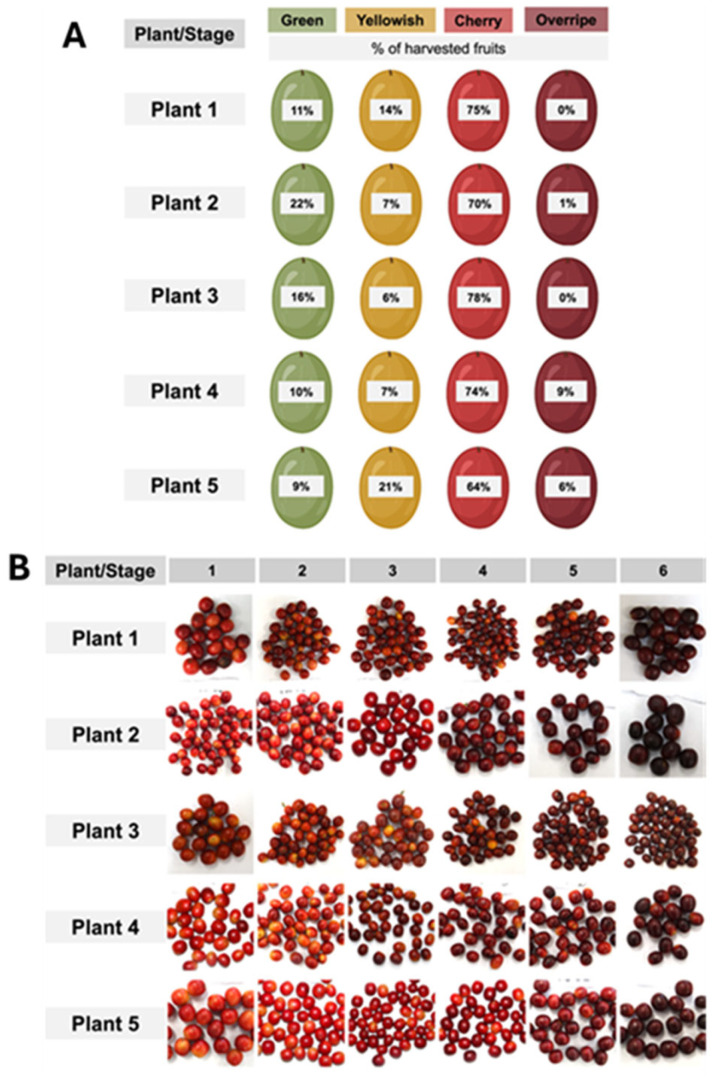
Percentage of fruits in four developmental stages (green, yellow, cherry, and overripe) in samples collected from five *C. arabica* plants (**A**) and distribution of the cherry fruits into six ripeness stages according to their redness intensity (**B**).

The fruits were separated into six classes of red, according to colourimeter measurements (Figure 1). The ripeness stages were divided arbitrarily into six groups based on the upper and lower boundary of each class, from light to dark red: Stage 1 (a* = +23.0 to +21.0), Stage 2 (a* = +20.9 to +19.0), Stage 3 (a* = +18.9 to +17.0), Stage 4 (a* = +16.9 to +15.0), Stage 5 (a* = +14.9 to +12.0), and Stage 6 (a* = +11.9 to +8.0).

**Figure 2:**
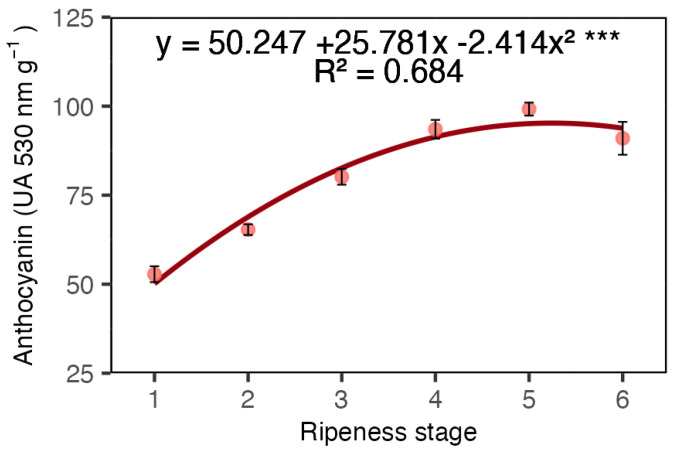
Anthocyanin concentration in Arabica coffee coat according to red ripeness classes. Data are means of five replicates and asterisks indicate curve significance at 0.001%. Bars at each ripeness stage indicate standard deviation.

The analysis of anthocyanin levels in the coffee coat revealed an increase up to the fifth stage, indicating a corresponding enhancement in the red colouration of the fruits (Figure 2), thus confirming the visual observation of increased redness depicted in Figure 1.

**Figure 3:**
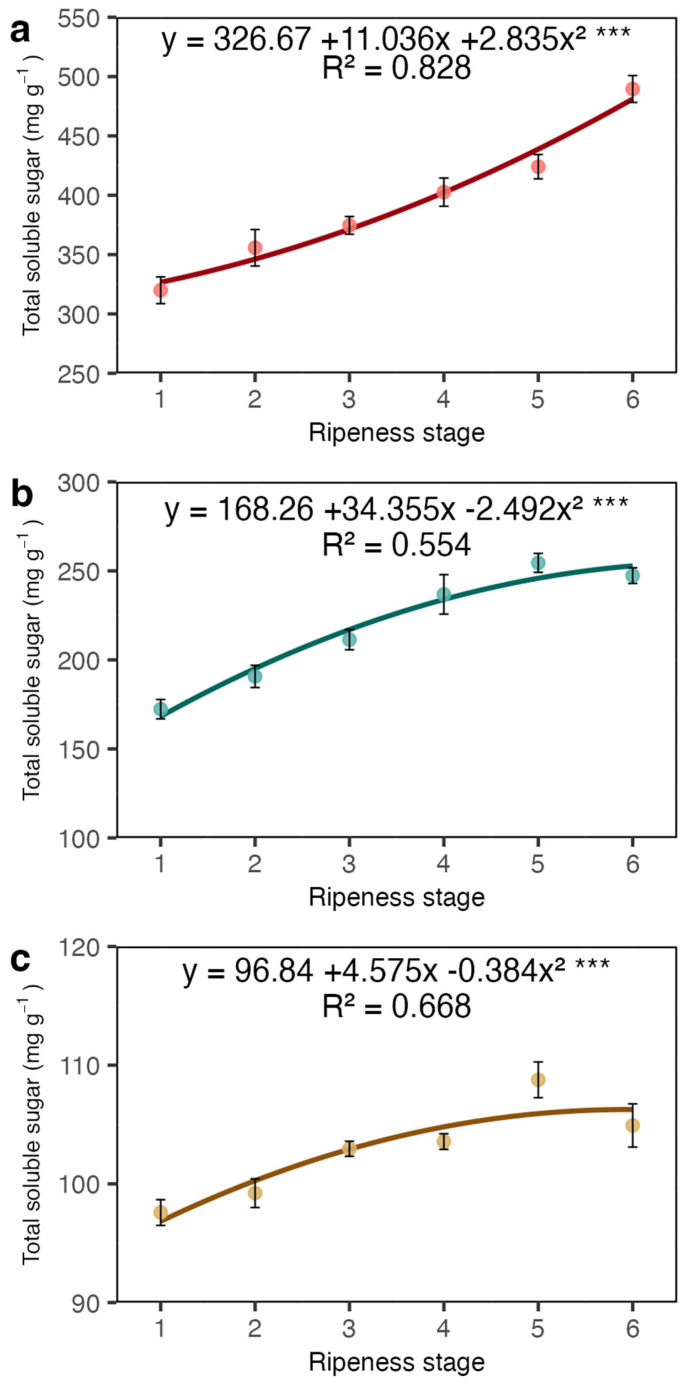
Total soluble sugar (TSS) concentrations in the coat (**a**), juice (**b**), and beans (**c**) of Arabica coffee according to the red ripeness classes. Data are means of five replicates and asterisks indicate curve significance at 1%. Bars in each ripeness stage indicate standard deviation.

The total soluble sugars (TSSs) increased in the coat, juice, and beans as maturation progressed and the redness changed (Figure 3). Remarkably, the coat exhibited the highest TSS content, while the beans had the lowest. Furthermore, the difference between the TSS levels at the initial and final ripeness stages was significantly higher in the coat, indicating the progression of fruit maturation.

**Figure 4:**
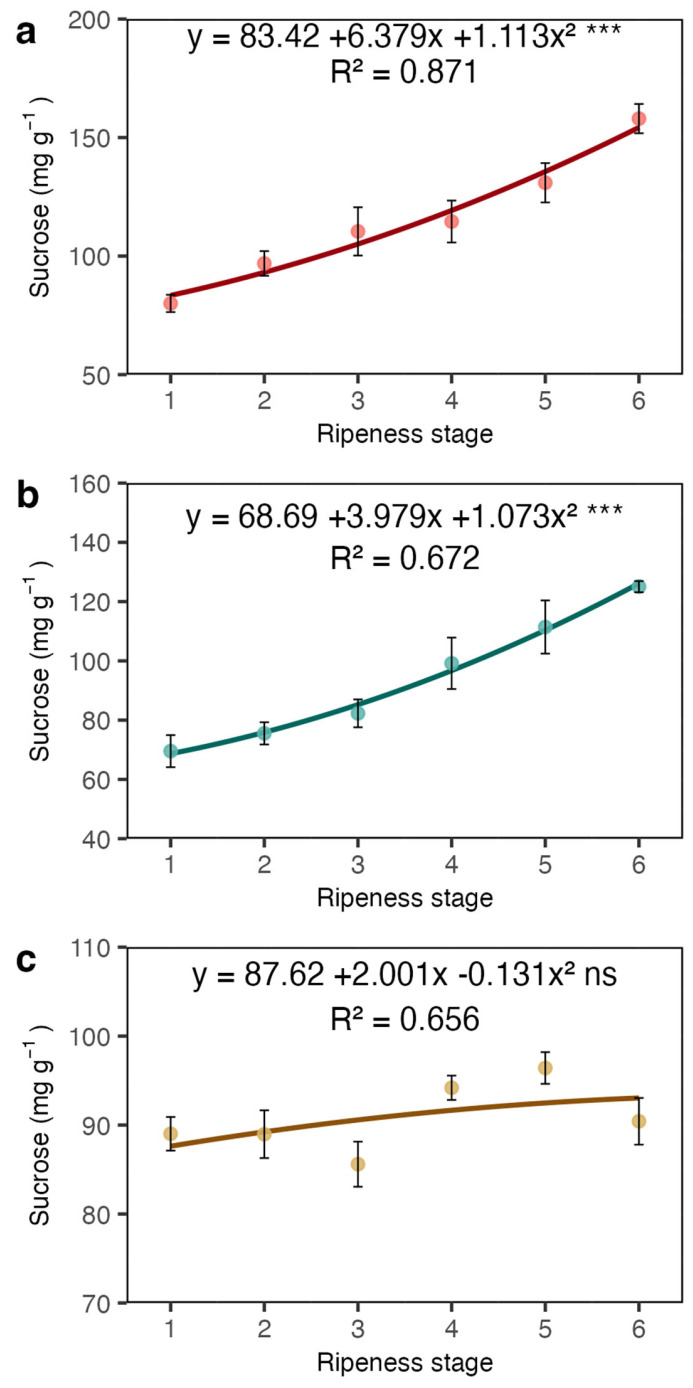
Sucrose concentrations in the coat (**a**), juice (**b**), and beans (**c**) of Arabica coffee according to the red ripeness classes. Data are means of five replicates and asterisks indicate curve significance at 1%. ns = not significant. Bars in each ripeness stage indicate standard deviation.

Sucrose, the primary soluble sugar present in coffee beans, exhibited a variation similar to TSSs in both the coat and juice. Specifically, sucrose concentration nearly doubled from stage 1 to stage 6 in both the coat and juice (Figure 4a,b). However, there was no significant variation in the beans, and sucrose concentration remained stable throughout the stages (Figure 4c).

**Figure 5:**
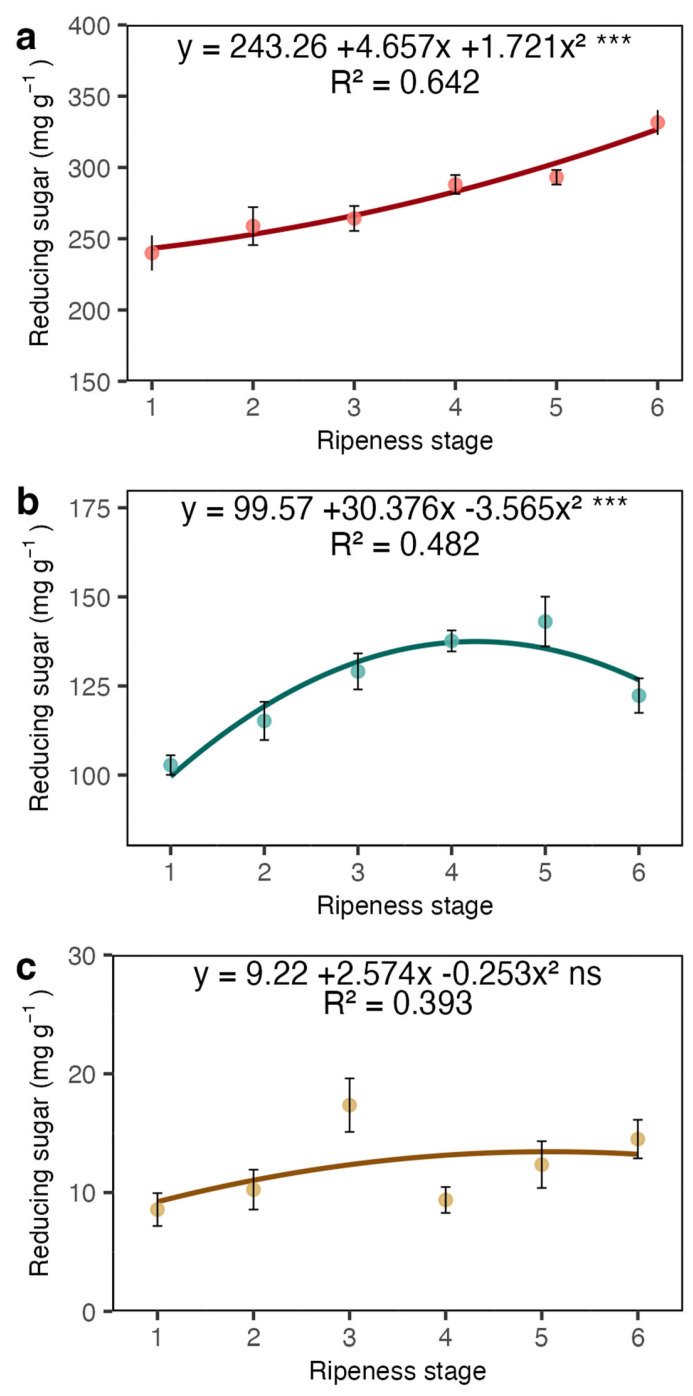
Reducing sugar concentrations in the coat (**a**), juice, (**b**) and beans (**c**) of Arabica coffee according to the red ripeness classes. Data are means of five replicates and asterisks indicate curve significance at 1%. ns = not significant. Bars in each ripeness stage indicate standard deviation.

The concentration of reducing sugars (RSs) in the coat increased, mirroring the pattern observed for TSSs and sucrose (Figure 5a). In contrast, in the juice, RSs reached their peak at stage 5 and then decreased at stage 6 (Figure 5b). Similar to sucrose, there were no significant changes in RS concentrations in the beans (Figure 5c).

**Figure 6:**
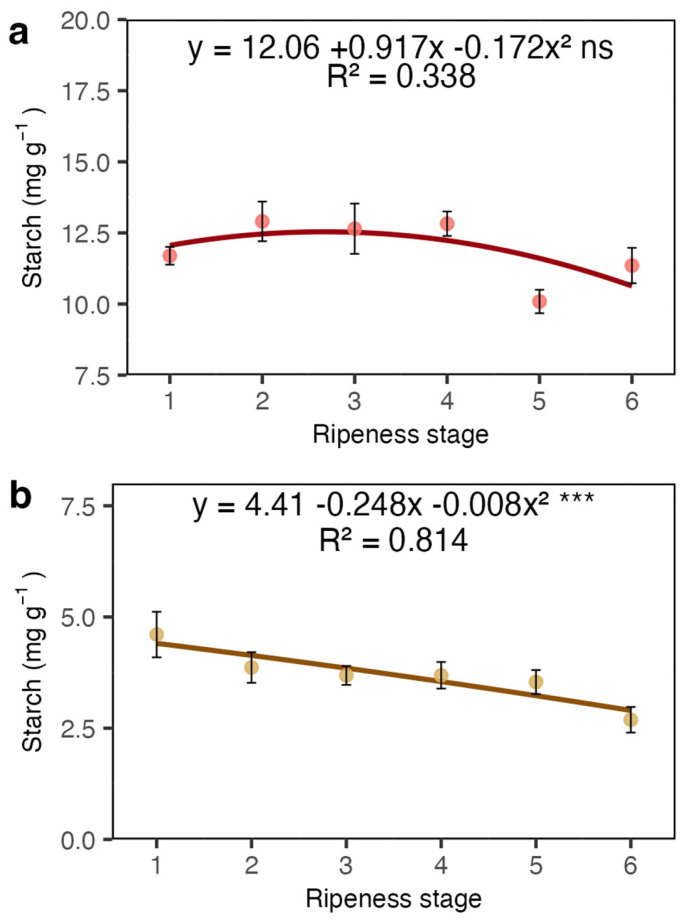
Starch concentrations in the coat (**a**) and beans (**b**) of Arabica coffee according to the red ripeness classes. Data are means of five replicates and asterisks indicate curve significance at 1%. ns = not significant. Bars in each ripeness stage indicate standard deviation.

Starch did not show significant variations in the coat (Figure 6a), but decreased in the beans as maturation progressed (Figure 6b). 

## 3. Discussion

The classification system we adopted to differentiate distinct shades of red was effective (Figure 1B). The darkening of the red colour in the fruit coat from stages one to six is attributed to the increase in anthocyanin (Figure 2). While there were visual similarities between adjacent ripeness stages (e.g., stages 1 and 2 or stages 2 and 3), the differences between other stages were distinguishable (Figure 1B). This suggests that if redness is associated with the ideal harvest stage, the variations in red hues could potentially be used to separate high-quality beans, thereby maximizing coffee cup quality.

If such colour variation occurs, one might wonder about the metabolic changes happening during these transitions. A previous study used a colourimeter to separate beans at different developmental stages and evaluate the metabolomic and proteomic changes [17]. Unfortunately, among the fruits at the four developmental stages, only two stages were red, and all metabolomic and proteomic analyses were conducted on whole fruits rather than separated tissues, limiting direct comparisons with our work.

A subsequent study used a colourimeter to study colour changes in eight classes of coffee fruit development, from green to dry fruits [5]. They analysed the fresh and dry biomasses of the peel, mucilage, and beans in fruits from these eight classes and used the classes M1, M2, and M3, corresponding to three red shades, for biochemical analysis of the beans, including sucrose, fructose, and glucose. The sucrose concentration in the beans decreased from 92.34 mg kg^−1^ (M1) to 86.09 mg kg^−1^ (M3). Fructose and glucose, which are reducing sugars, were found in minor amounts, ranging from 0.36 mg kg^−1^ (M1) to 0.62 mg kg^−1^ (M3) and from 0.19 mg kg^−1^ (M1) to 0.34 mg kg^−1^ (M3), respectively. The sucrose concentration was in the same range as we found here, but not reducing sugars, as the method we used here detects all reducing sugars and does not differentiate glucose from fructose.

Coffee fruits increase in volume and dry mass during maturation, which occurs in the second growth phase of the bimodal growth curve of coffee beans. During this phase, there is a notable increase in the growth rate of the pericarp. Meanwhile, the beans cease growing and reach their maximum size and dry mass during the first growth phase [9,12]. Sucrose concentrations continue to increase throughout fruit growth, unlike other compounds such as caffeine, trigonelline, proteins, and chlorogenic acids, which reach their peak concentrations in the first growth phase [10,18], also known as the storage phase [19]. Transcriptomic analysis also revealed that most genes associated with seed reserves are expressed during the transition from green (with a fully formed endosperm) to yellow (beginning of maturation) fruits, with 2058 differentially expressed transcripts (both upregulated and downregulated). In contrast, the comparison between the red (mature) and yellow stages showed only 130 differentially expressed genes [20].

This increase was investigated by feeding coffee fruits or leaves on branches bearing fruits with ^14^CO_2_ [9]. The ^14^C-sucrose, the product of photosynthesis in the fruits and leaves, was found in high concentrations in the coat of fruits where ^14^CO_2_ was supplied to the leaves. However, about 30–40% of the total radioactivity found in the fruit was in the endosperm and perisperm, indicating that sucrose from leaves is imported into various parts of the fruit. When the fruits were incubated with ^14^CO_2_, most radioactivity was found in the perisperm, suggesting that this tissue acts as a conduit. Indeed, the perisperm is connected to the pericarp by a kind of bridge formed by a few cells [9]. However, the endosperm is not directly connected to any other fruit tissue, implying that molecules transported from the leaf or the coat photosynthesis to the endosperm follow an apoplastic pathway [9]. 

Our results showed a sharp increase in total soluble sugars and sucrose in the coat and juice (the liquid obtained from squeezed coats). This increase in sucrose may be related to the high respiration rate occurring in fruits during maturation [21] and is likely imported from the leaves [9]. However, most of the total sugars were represented by reducing sugars (Figure 3a and Figure 5a), which may have resulted from the degradation of pectin in the mesocarp. The sugars detected in the juice were certainly a rich fraction of these reducing sugars. The coffee pericarp is rich in pectin [22], and during maturation, there is an increase in enzyme activity related to pectin degradation, such as pectinase and pectin lyase [20,23]. This process is accompanied by an increase in the concentration of reducing sugars like arabinose and galactose, among others. Given the low amount of starch in the coat and endosperm (Figure 6a,b), its contribution to the sugar content seems very limited.

It is well known that while sucrose increases in the endosperm, reducing sugars like fructose and glucose decrease [9,13,24]. In our study, both sucrose (Figure 4c) and reducing sugars (Figure 5c) in the endosperm remained stable. Thus, despite a marked increase in soluble sugars in the coat, which includes both sucrose and reducing sugars, these sugars do not appear to be transferred to the endosperm. We suggest that the endocarp, which is primarily composed of cellulose (40–49%), hemicellulose (25–32%), and lignin (33–35%) and is relatively impermeable [25], may act as a barrier to the transport of sugars from the coat to the endosperm. Due to its lignocellulosic composition, the endocarp (parchment in dry seeds) has a low hygroscopic nature, which offers resistance to water transfer [26].

Although the increase in sucrose was not pronounced in the endosperm across the six red classes we identified, it would be worthwhile to test the beverage quality to determine if such a discrete increase might affect the overall quality.

Identifying the optimal ripeness stage is crucial for determining the best harvest timing and enhancing coffee quality. However, depending on the size of the coffee farm, harvesting can become a logistical challenge if maturation occurs homogeneously across the entire farm. To address this, selecting varieties with different maturity groups can help stagger the harvest into a more controlled process, thus avoiding the collection of green fruits. Additionally, remote sensing and colour sorting machines can assist in identifying the ideal ripeness stage of the coffee crop and sorting the fruits by colour after harvest, resulting in more uniform and high-quality coffee lots. Ultimately, these practices would lead to better products in the market, increasing the profitability and sustainability of coffee cultivation.

## 4. Materials and Methods

### 4.1. Experimental Design and Fruit Harvest

The samples were collected from coffee trees (“Red Obatã” variety) in the 2017/2018 season in the experimental area of “Luiz de Queiroz” College of Agriculture–Esalq/Usp at Piracicaba-SP. Fruits were manually harvested from five six-year-old coffee trees. The branches collected were in the mid-third of the plants. In the lab, the fruits were visually classified into green (unripe fruits), yellowish (moderately ripe), cherry (completely red fruits), and overripe fruits. As indicated in Figure 1, most of the fruits were at the red stage, and only these fruits were used for further analysis. The fruits were processed and analysed immediately after classification by colour, as indicated in Section 4.2.

### 4.2. Fruit Classification by Colour

Two hundred red fruits were selected randomly from each plant for colour classification and were classified separately by colour, representing five replicates. The colour was measured by placing each fruit laterally on a Minolta CR300 colourimeter (Konica Minolta, Tokyo, Japan), which measures colour using the CIELAB pattern (International Commission on Illumination). In this system, colour is classified by the L*, a*, and b* coordinates in a three-dimensional space, where the first axis, L*, is the lightness of the colour and varies from 0 (black) to 100 (white), while the other two axes, a* and b*, indicate the relative coordinates of the colour across red, green, blue, and yellow. The a* coordinate is the position between red (positive values) and green (negative values), and the b* coordinate is the position between blue (positive values) and yellow (negative values). All three parameters were obtained, but only the a* parameter (coordinate red/green) was used to classify the fruits into six ripeness stages based on the red colour. 

### 4.3. Sample Preparation and Analysis

Each fruit’s coat (epicarp and mesocarp) was manually removed and then manually squeezed into tubes maintained on ice to extract the juice from the mesocarp. The fruits were squeezed several times to remove the juice completely. We estimated the volume of the juice in a 10 mL cylinder, quickly centrifuged at 4 °C, and then immediately froze the supernatant at −20 °C. The coat was also frozen. The beans were rubbed in a cotton tissue to remove the adherent mucilage, and the hard parchment (endocarp) was manually removed and discarded. The beans were frozen, too. The frozen coat and beans were lyophilized and finally grounded in a mortar with a pestle.

We determined total soluble sugars, sucrose, reducing sugars (subtracting sucrose from total soluble sugars) and starch content in all samples, except for the juice, where starch was not determined. Coat and beans were processed with 70% ethanol (100 mg/1 mL of) at 80 °C for 10 min. The samples were centrifuged at 10,000× *g* for 10 min, the supernatant was collected, and the precipitate was re-extracted five more times in 70% ethanol. The six extractions were combined. The remaining residue was used for starch determination, using 1 mL of 30% perchloric acid [27]. The samples were left resting overnight on the bench, and the supernatant was recovered by centrifugation and used to determine glucose residues using the phenol sulfuric method [28]. Briefly, 0.5 mL sample (or less, but completing the final volume to 0.5 mL with the extraction solvent) was mixed with 0.5 mL phenol reagent, and 2 mL of concentrated H_2_SO_4_. After vortexing, the tubes were left to cool down on the bench and then the absorbance was read at 490 nm. As a blank, we made a reaction using the extraction solution instead of the sample. Glucose was used as a standard to produce a calibration curve. Total soluble sugar was determined using the phenol sulfuric method [28] and sucrose was used as a standard to produce a calibration curve. Sucrose in the extracts was determined after hydrolysis of the reducing sugars with 30% KOH incubation at 100 °C, as proposed by Van Handel [29]. The samples were mixed with an equal volume of aqueous 30% KOH, vortexed, and incubated in a water bath at 100 °C for 10 min. After cooling down on the bench, the samples had their sucrose concentrations determined using the phenol sulfuric method. Sucrose was used as the reference standard to produce a calibration curve. The juice was used for analysis of sucrose and total soluble sugars without any previous extraction. Reducing sugar concentration was obtained based on the difference between total soluble sugars and sucrose determinations. Anthocyanin was also estimated in the coat [30]. Briefly, extraction was done by adding 1 mL of a solution containing 6 M HCl, water, and methanol (7:13:70, *v*/*v*/*v*) to 100 mg grounded samples, followed by incubation at 4 °C for 24 h in the dark. Next, the samples were centrifuged at 14,000 rpm for 10 min, and the supernatant was collected and analysed in a spectrophotometer at two wavelengths (530 and 653 nm). The anthocyanin concentration was calculated using the formula C = A530 − (0.24 × A653), where C is the anthocyanin concentration in absorbance units; A530 is the absorbance at 530 nm; and A653 is the absorbance at 653 nm. The amount of anthocyanin was expressed as the calculated value divided by the tissue mass used in the extraction.

### 4.4. Data Analysis

All data analyses were conducted using R software (version 4.2.2), and all plots were generated using the ggplot2 package. We used linear mixed models (package lme4), setting the coffee ripeness stage as the fixed effect and the plants (replicate) as random factors for all variables. Analysis of variance was conducted using the car package.

## Data Availability

Data are available upon request to the corresponding author.
